# Caffeic Acid–Chicoric Acid (1:1) Mixture Exerts Antioxidant Effects and Regulates Intestinal Health in Oxazolone-Induced Zebrafish

**DOI:** 10.3390/antiox15040419

**Published:** 2026-03-27

**Authors:** Weiwei Zhou, Xuefeng Wang, Zufeng Zhang, Wei Tian, Jinhua Zhao, Xiumei Li

**Affiliations:** 1 Institute of Feed Research of Chinese Academy of Agricultural Sciences, Beijing 100081, China; zhouweiwei@caas.cn (W.Z.); young7854@live.hccc.edu (X.W.); zhangzufeng@mail.tust.edu.cn (Z.Z.); 1063901698@mail.tust.edu.cn (W.T.); zhaojinhua@caas.cn (J.Z.); 2 College of Biotechnology, Tianjin University of Science and Technology, Tianjin 300457, China

**Keywords:** caffeic acid, chicoric acid, oxidative stress, molecular mechanism, intestine microbiota

## Abstract

Caffeic acid (CaA) and chicoric acid (ChA) each exhibit significant antioxidant activity when used alone, yet their combined effects on antioxidant capacity and intestinal health in zebrafish remain unclear. This study used isobolographic analysis to identify a 1:1 ratio of CaA to ChA as optimal for synergistic antioxidant activity, with its ABTS and DPPH IC_50_ (21.65 μg/mL, 69.66 μg/mL) outperforming single monomers in vitro. In an oxazolone (Oxa)-induced zebrafish intestinal oxidative stress model, the CaA and ChA (CaA–ChA) mixture exerted antioxidant effects by upregulating the mRNA expression levels of *HTR2A*, *Akt*, *Nrf2*, and downstream antioxidant enzyme genes including *SOD*, *CAT*, and *GPx* in the zebrafish intestine, while downregulating *Keap1* mRNA expression. Intestinal microbiota analysis revealed that the CaA–ChA mixture could positively regulate the intestinal microecological structure, characterized by targeted enrichment of the beneficial bacterium *Cetobacterium* and inhibition of the proliferation of potential pathogenic bacteria, including *Bosea* and *Mycobacterium*. Correlation analysis confirmed that the abundances of these key genera were closely associated with the expression of signaling pathway markers, suggesting that the microbiota–signaling pathway crosstalk was involved in the regulation of antioxidant processes. In conclusion, the CaA–ChA mixture (1:1) exerts a protective effect against intestinal oxidative stress, with the potential involvement of dual gut microbiota modulation and the HTR2A/Akt/Nrf2/Keap1 pathway. These findings provide experimental and theoretical support for the combined antioxidative application of CaA and ChA.

## 1. Introduction

Oxidative stress is a state of imbalance in the body’s redox equilibrium caused by a variety of pathological factors. Oxidative stress can trigger intestinal diseases, such as inflammatory bowel disease and irritable bowel syndrome, by disrupting the physiological functions of biological macromolecules such as DNA, proteins, and lipids [1,2]. The intestine is constantly exposed to stimuli from food residues and microorganisms, making it highly susceptible to oxidative stress [3]. Oxidative stress in the intestine is closely related to the metabolic imbalance of reactive oxygen species (ROS). ROS, by-products of mitochondrial respiration, play a regulatory role in physiological functions at normal levels [4]. However, when the body’s ROS exceed the clearance capacity, it can lead to oxidative damage to biological macromolecules, disrupt the intestinal barrier, and cause inflammatory responses [5]. The physiological processes mediated by inflammatory factors will provide free radicals, which promote the progression of oxidative stress, exacerbating it [6].

A substantial amount of research has demonstrated that polyphenolic compounds possess significant antioxidant properties [7,8]. Among them, caffeic acid (CaA), with the molecular formula C_9_H_8_O_4_, is readily soluble in hot water and ethanol. Studies indicated that CaA had antioxidant and anti-inflammatory activities, and its mechanism of action was complex and diverse [9]. Concretely, CaA could provide electrons and hydrogen atoms to free radicals, converting them into non-radical products. Free radicals had a conjugation effect with the aromatic ring, and the electrons of the product were dispersed within the aromatic ring of CaA, making the product more stable and inhibiting the free radical chain reaction [10]. CaA also acted as a chelating agent in the body, forming complexes with metal ions to inhibit the decomposition of peroxides and reduce the formation of free radicals, thereby mitigating their destructive effects on biological macromolecules and protecting cellular integrity [11]. CaA also had a regulatory effect on antioxidant-related enzyme systems. It was reported that CaA could significantly improve the oxidative stress status and protect neural functions in SD rats with cerebral ischemia. The mechanism might involve the activation of the Nrf2 signaling pathway by CaA, leading to increased expression of glutathione peroxidase and decreased expression of iron ion oxidoreductases [12,13].

Chicoric acid (ChA), with the molecular formula C_22_H_18_O_12_, typically exists in nature as the levorotatory form, and its structure is formed by the dehydration condensation of two molecules of CaA. It is readily soluble in organic solvents such as methanol and ethanol [14]. As a derivative of CaA, ChA also possess antioxidant activity. Studies found that ChA had a protective effect against oxidative damage to proteins, lipids, and DNA induced by reactive oxygen species. Xiao Haifeng et al. [15] used ChA to pre-protect bovine serum albumin, mouse liver brain protein, and herring sperm DNA and found that ChA could significantly reduce the oxidative degradation of proteins and decrease the production of carbonylated proteins. At the same time, it significantly reduced the level of lipid peroxides in tissues and the degree of DNA degradation [16]. In addition, ChA showed good protective effects against oxidative damage to biological macromolecules caused by metal-catalyzed oxidation and superoxide radicals. Li Yipeng et al. [17] extracted ChA from the roots of *Echinacea purpurea* and applied it to the oxidative stress models of Caco-2 cells and porcine intestinal epithelial cells (IPEC-J2), finding that ChA had good protective effects on both types of cells, and the effects were dose-dependent. Kim et al. [18] found *Crepidiastrum denticulatum* extract and its active compound ChA could upregulate antioxidant enzymes and decrease inflammation by inhibiting proinflammatory cytokines and nuclear factor-κB activation. ChA could also reduce neuronal apoptosis and improve cognitive impairments caused by oxidative stress in the brain [19].

Our research group previously demonstrated that, at the cellular level, the extract of *Sonchus brachyotus* DC. exerted a significant reparative effect on oxidative damage and elucidated its antioxidant mechanism through the Nrf2–Keap1–ARE signaling pathway. In the oxazolone (Oxa)-induced zebrafish model of intestinal oxidative stress injury, this extract conferred a protective effect against oxidative stress by regulating the crosstalk between oxidative stress biomarkers and the intestinal microbiota. Additionally, our preliminary studies identified that the extract of *Sonchus brachyotus* DC. was abundant in CaA and ChA [20,21]. It has been reported that both phytochemicals can effectively ameliorate the oxidative stress status in organisms and enhance the endogenous antioxidant capacity of the body. However, no relevant reports on their synergistic antioxidant effects have been published to date [22,23].

Based on the above research foundation, the present study first verified the synergistic antioxidant effect of CaA and ChA in vitro. Furthermore, according to the actual scavenging activities of CaA, ChA, and their combinations (CaA–ChA) against ABTS and DPPH free radicals, isobologram analysis was employed to determine the optimal ratio of the two compounds for exerting the maximum antioxidant activity. This study further investigated the antioxidant mechanism of the CaA–ChA mixture on the basis of in vitro experimental results. Specifically, network pharmacology approaches were first adopted to screen the core targets and key signaling pathways mediating the antioxidant effect of the CaA–ChA combination. Subsequently, based on the Oxa-induced zebrafish oxidative stress model, we detected the effects of this combination on the expression of core target genes, the activity of antioxidant enzymes, the expression of tight junction proteins, and alterations in the intestinal microbial community structure in the zebrafish intestine, so as to validate its antioxidant mechanism at the in vivo level. Finally, the synergistic antioxidant mechanism of the CaA–ChA combination was initially elucidated by analyzing the correlation between oxidative-stress-related biomarkers and the relative abundance of intestinal microbiota. This study not only clarifies the antioxidant mechanism of CaA and ChA but also supplements and improves the research system of *Sonchus brachyotus* DC. in the field of antioxidation, thus possessing important academic value and research significance.

## 2. Materials and Methods

### 2.1. Chemicals and Reagents

CaA (purity: HPLC ≥ 98%), ChA (purity: HPLC ≥ 98%), and DPPH-free-radical-scavenging-ability test kits were purchased from Solarbio Technology Co., Ltd. (Beijing, China). The ABTS-free-radical-scavenging-ability test kit was purchased from Beyotime Biotechnology Co., Ltd. (Shanghai, China). Isopropanol chloroform, anhydrous ethanol, and Trizol reagent were purchased from Sinopharm Chemical Reagent Co., Ltd. (Shanghai, China). FastReal qPCR PreMix (SYBR Green) was purchased from Tiangen Biotech Co., Ltd. (Beijing, China). TransScript R First-Strand cDNA Synthesis SuperMix was purchased from TransGen Biotech Co., Ltd. (Beijing, China). Oxa was purchased from Sigma-Aldrich Co., Ltd. (Shanghai, China). All the primers were purchased from Sangon Biotech Co., Ltd. (Shanghai, China).

### 2.2. Sample Preparation

CaA and ChA were weighed to 5 mg and dissolved in 50 μL of methanol, respectively. Then, water was added to obtain a 5 mg/mL stock solution. The stock solution was diluted with distilled water to prepare solutions with concentrations of 2.5, 5, 10, 12.5, 20, 25, 40, 50, 60, 80, 100, 120, 150, 200, 300, 400, and 500 μg/mL for CaA and ChA, respectively. Finally, CaA and ChA were mixed in a ratio of 1:2, 1:1, and 2:1 for the determination of free-radical-scavenging ability.

### 2.3. Determination of ABTS and DPPH Free-Radical-Scavenging Activity

The DPPH and ABTS assays were used to assess the antioxidant capacity of samples [24]. The ABTS stock solution was prepared according to the kit instructions by mixing the oxidizer with the ABTS solution at a 1:1 ratio and then diluted with distilled water until the absorbance was 0.7 ± 0.05 at 734 nm to obtain the ABTS free-radical-scavenging working solution. The various concentrations of samples were added to the 96-well plate. Three replicates were set up for each sample with 10 μL per well; then, the ABTS working solution was added and incubated at room temperature for 6 min, and the absorbance was measured at 734 nm with a microplate reader. The ABTS scavenging rate was calculated as follows: [(A blank − A sample)/A sample] × 100%.

The DPPH powder was diluted from the kit with anhydrous ethanol to prepare the DPPH stock solution. The stock solution was mixed with anhydrous ethanol in a ratio to prepare the working solution. Then, 5 μL of the sample solution was added to the test wells and the color control wells, and 5 μL of distilled water was added to the blank control wells. Subsequently, 195 μL of DPPH working solution was added to the blank control wells and the test wells, and 195 μL of anhydrous ethanol was added to the color control wells. Three replicates were set up for each sample. Finally, the kit was placed at room temperature in the dark for 30 min, and the absorbance was measured at 515 nm. The DPPH free-radical-scavenging rate (%) was calculated as follows: [[A blank − (A measure − A control)]/A blank] × 100%.

### 2.4. Isobolographic Analysis of Synergistic Antioxidant Capacity

In this experiment, the equivalent index of synergistic antioxidant capacity was set as the concentration of the antioxidant required to eliminate half of the free radicals, namely the half-maximal scavenging concentration (IC_50_) [25]. Initially, the free-radical-scavenging rates of various concentrations of CaA, ChA, and CaA–ChA on ABTS and DPPH radicals were measured. Subsequently, based on the measured free-radical-scavenging rates, dose–effect curves were plotted, and the curves were linearly analyzed to calculate the IC_50_ of CaA, ChA, and their mixtures with GraphPad Prism 8.0 software.

The potency ratio of CaA to ChA, was represented by R ChA. The calculation formula was R ChA = IC_50_ CaA/IC_50_ ChA. Assuming an additive effect between CaA and ChA, the theoretical value of the IC_50_ add when used in combination could be calculated using the equation IC_50_ add = IC_50_ CaA/(K CaA + R ChA × K ChA), where K CaA and K ChA represented the proportions of CaA and ChA in the mixture, respectively. Then, the 95% confidence intervals for the IC_50_ of CaA and ChA were calculated. The criteria for evaluating synergistic effects were as follows: if the IC_50_ add was approximately equal to the IC_50_ mix, meaning both the IC_50_ mix and the IC_50_ add fall within the additive line or 95% confidence interval, the interaction between the substances was not significant, and the combination of CaA and ChA exhibited an antioxidant additive effect. If the IC_50_ add was significantly lower than the IC_50_ mix, meaning that the IC_50_ mix was above the upper limit of the 95% confidence interval of the additive line, it indicated an antagonistic effect in antioxidant activity. If the IC_50_ add was significantly higher than the IC_50_ mix, meaning that the IC_50_ mix was below the lower limit of the 95% confidence interval of the additive line, it suggested that CaA and ChA had a synergistic antioxidant effect. The strength of the synergistic effect between CaA and ChA was represented by the γ value, with the calculation formula being as follows: γ = IC_50_ CaA mix/IC_50_ CaA + IC_50_ ChA mix/IC_50_ ChA, where IC_50_ of CaA mix and IC_50_ of CaA mix referred to the IC_50_ values of CaA and ChA, respectively, within the combined system of the two antioxidants. A γ value less than 1 indicated the presence of synergy between the two, with a lower γ value indicating a stronger synergistic effect between CaA and ChA [26].

### 2.5. Network Pharmacology Analysis

Network pharmacology was performed to preliminarily explore the relevant key targets and pathways of CaA–ChA action [27,28]. The action targets of CaA and ChA were retrieved from the online TCMSP database (https://old.tcmsp-e.com/tcmsp.php, accessed on 19 March 2026), the Herb database (http://herb.ac.cn/, accessed on 19 March 2026), and the BATMAN-TCM database (http://bionet.ncpsb.org.cn/batman-tcm/index.php, accessed on 19 March 2026). Also, the 3-dimensional structures of CaA and ChA were obtained from PubChem (https://pubchem.ncbi.nlm.nih.gov/, accessed on 19 March 2026) and were subjected to the Swiss Target Prediction database (http://www.Swisstargetprediction.ch, accessed on 19 March 2026) for the prediction of related targets. The oxidative stress-related disease targets were collected from GeneCards database and OMIM database. All the targets were integrated, removing the duplicates, and standardized using the UniProt database. Wayne analysis was used to obtain the intersection targets of action for CaA, ChA, and oxidative -stress-related disease targets. Then, the intersection targets were subjected to Cytoscape (v.3.7.2) to construct the “CaA and ChA-antioxidant-target” network.

The protein–protein interaction (PPI) network was generated by inputting the targets into the online STRING database (https://string-db.org/, accessed on 19 March 2026). Based on the retrieval of interactive targets, the Gene Ontology (GO) enrichment analysis, including biological process, cellular component, and molecular function, and the Kyoto Encyclopedia of Genes and Genomes (KEGG) enrichment analysis were obtained using the Database for Annotation, Visualization and Integrated Discovery (DAVID) (https://david.ncifcrf.gov/home.jsp, accessed on 19 March 2026). The results of the enrichment analysis were visualized using Bioinformatics (http://www.bioinformatics.com.cn/, accessed on 19 March 2026).

### 2.6. Animal Experiments

Adult zebrafish were randomly divided into 6 groups (30 fish per group): a blank control group (Con), a solvent control group (EtOH), a model group (Oxa), a CaA treatment group, a ChA treatment group, and a CaA–ChA treatment group. The control group and the model group were fed with a drug-free basal diet, while each treatment group was separately fed with the diet supplemented with 0.03 mg/kg CaA, 0.03 mg/kg ChA, and 0.03 mg/kg CaA–ChA (0.03 mg/kg CaA + 0.03 mg/kg ChA), respectively, for a consecutive period of 14 days. After the final feeding, all experimental fish were subjected to a 12 h fasting period. Subsequently, the zebrafish intestinal oxidative stress model was induced using 0.2% Oxa with ethanol as the solvent, as described by Yang et al. [21]. After the last feeding, the zebrafish were fasted for 24 h and anesthetized with a 0.03% solution of tricaine methanesulfonate solution. The blank control group was injected with 0.9% physiological saline, the model group and treatment groups were injected with 0.2% Oxa, and the solvent control group was injected with 50% ethanol, with an injection volume of 0.6 μL/100 mg for each. After a 24 h fasting period, the zebrafish intestines were collected for subsequent testing.

### 2.7. Quantitative Real-Time PCR

Zebrafish intestinal tissue (0.1 g) was weighed in a centrifuge tube, and 1 mL of Trizol lysis buffer was added and lysed on ice for 5 min. After completion, the RNA containing lysis buffer was mixed and collected in a 1.5 mL centrifuge tube. According to the kit instruction, the total RNA from cell and tissue samples was extracted, and the cDNA was obtained through reverse transcription. PCR was performed according to the procedure (10 min at 95 °C, 15 s at 95 °C, 1 min at 60 °C, amplification for 40 cycles, 15 s at 95 °C) with the reaction system of 2 µL of cDNA, 0.5 µL of upstream primers, 0.5 µL of downstream primers, 5 µL of Power SYBR Green PCR Master Mix, and 7 µL of ddH_2_O [29]. The primer sequences used are shown in Table 1.

### 2.8. Histological Analysis

Zebrafish intestinal tracts were harvested, washed, and embedded in 4% paraformaldehyde. Then, the fixed tissue samples were dehydrated with gradient ethanol or acetone. Xylene was used to completely replaced the water in the tissue in preparation for embedding. The clarified tissue samples were embedded in paraffin wax so that they could be cut into thin sections. The sections were stained with hematoxylin–eosin (H&E) staining to highlight different structures of tissues. Finally, the sections were placed under a microscope to observe and analyze the morphology, arrangement, and structure of cells and tissues. The images of the microscopic observations were captured for further analysis and documentation [30,31].

### 2.9. 16S rDNA Gene Sequencing

Bacterial DNA was collected from the intestinal contents of zebrafish, and PCR amplicon sequencing and gene analysis were conducted by Biozeron Biotechnology Co. Ltd. (Shanghai, China). Based on the PacBio sequencing platform, a single-molecule real-time sequencing (SMRT Cell) method was used to sequence the marker genes, followed by CCS (Circular Consensus Sequencing) sequence filtering to obtain Optimization-CCS for OTU (operational taxonomic unit) clustering, species annotation, and abundance analysis. Barcoded specific primers were synthesized based on the full-length 16S primers 27F (5′-AGRGTTTGATYNTGGCTCAG-3′) and 1492R (5′-TASGGHTACCTTGTTASGACTT-3′) for PCR amplification. The products were purified, quantified, and homogenized to form a sequencing library (SMRT Bell), which was first quality-checked and sequenced by PacBio Sequel. Subsequently, the differences between samples were explored through the analysis of α diversity and β diversity at the phylum and genus levels, as well as significant species difference analysis [32].

### 2.10. Correlation Analysis

The microbial diversity analysis was performed using Kindstar Online Platform (http://cloud.kindstarseq.com:8000/#/login, accessed on 19 March 2026). The correlation analysis was performed using a Spearman rank correlation analysis, and the significances were calculated at *p* ≤ 0.05 [33,34,35].

### 2.11. Statistical Analysis

The experimental data of this study and experimental graphing were statistically analyzed and processed using GraphPad Prism 8.0 software. Results were presented as the mean ± SD. Statistical analyses were performed using one-way analysis of variance (ANOVA) followed by the Least Significance Difference (LSD) test when more than two groups were compared. A *p* value of <0.05 was considered statistically significant.

## 3. Results

### 3.1. Determination of the Optimal Ratio of CaA and ChA with Antioxidant Effect

#### 3.1.1. The Free-Radical-Scavenging Ability of CaA and ChA

**Figure 1:**
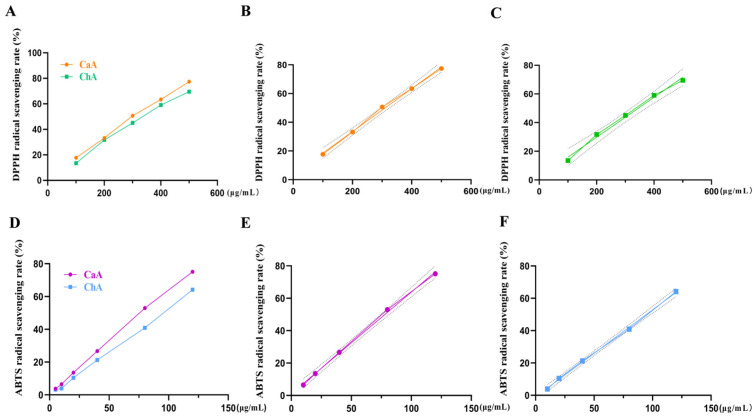
Free-radical-scavenging rates of CaA and ChA, respectively. (**A**) DPPH radical-scavenging rate of CaA and ChA. (**B**) Dose–effect curve of CaA based on DPPH radical-scavenging rate. (**C**) Dose–effect curve of ChA based on DPPH radical-scavenging rate. (**D**) ABTS radical-scavenging rate for CaA and ChA. (**E**) Dose–effect curve of CaA based on ABTS radical-scavenging rate. (**F**) Dose–effect curve of ChA based on ABTS radical-scavenging rate.

The free-radical-scavenging ability and dose–response relationship of CaA and ChA against DPPH radicals are shown in Figure 1A–C. As shown in Figure 1A, within the concentration range of 100 to 500 μg/mL, the DPPH free-radical-scavenging rate of CaA was consistently higher than that of ChA. As shown in Figure 1B,C, the IC_50_ of CaA and ChA was calculated using the fitted linear regression equation (R^2^ > 0.99), with IC_50_ values of 310.2 μg/mL and 344.8 μg/mL, respectively. Linear analysis indicated that the scavenging ability of CaA and ChA towards DPPH radicals was positively correlated with their concentration.

The free-radical-scavenging ability and dose–response relationship of CaA and ChA against ABTS radicals are shown in Figure 1D–F. As shown in Figure 1D, within the concentration range of 3.75 to 120 μg/mL, the ABTS free-radical-scavenging rate of CaA was consistently higher than that of ChA. As shown in Figure 1E,F, the IC_50_ values for CaA and ChA were 78.03 μg/mL and 94.77 μg/mL, respectively, and the scavenging abilities of CaA and ChA towards ABTS radicals were positively correlated with their concentration.

#### 3.1.2. The Free-Radical-Scavenging Ability of CaA–ChA

The CaA and ChA aqueous solutions were prepared according to the method described in Section 2.2, and they were mixed in proportion to obtain mixture solutions with different ratios and gradients. The free-radical-scavenging abilities of the CaA–ChA against DPPH and ABTS radicals are shown in Table 2 and Table 3, respectively. By plotting the concentration of the component with a lower proportion between CaA and ChA as the horizontal axis and the free-radical-scavenging rate as the vertical axis, linear fitting was performed, and the IC_50_ for the DPPH radicals was calculated to be 105.3 μg/mL, 69.66 μg/mL, and 111.4 μg/mL for the mixture ratios of 1:2, 1:1, and 2:1 of CaA to ChA, respectively. For the ABTS radicals, the IC_50_ values were 30.34 μg/mL, 21.65 μg/mL, and 27.85 μg/mL, respectively. It could be observed that the mixture of CaA and ChA significantly enhanced the scavenging ability against DPPH and ABTS radicals compared to when used individually, and the 1:1 mixture ratio had a lower IC_50_ for both DPPH and ABTS radicals compared to the other mixture ratios.

#### 3.1.3. Isobolographic Analysis of Synergistic Antioxidant Effects of CaA–ChA

**Figure 2:**
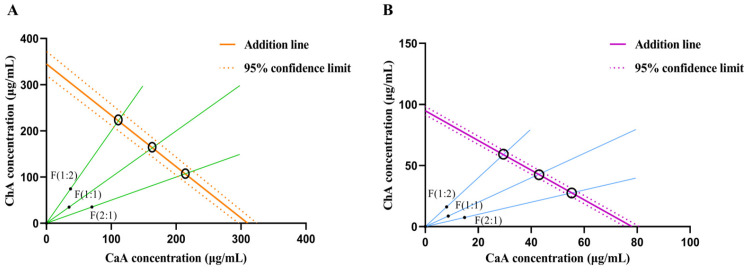
Isobolographic analysis diagram of scavenging rate of CaA–ChA. (**A**) DPPH. (**B**) ABTS. The green and blue lines represent isoboles, which depict the concentration combinations of CaA and ChA required to achieve a fixed antioxidant effect.

As shown in Figure 2A,B, the effects of CaA–ChA at different mixing ratios were all located below the lower limit of the 95% confidence interval of the additive line, indicating that CaA–ChA at various ratios exhibited the ability to synergistically scavenge DPPH and ABTS free radicals.

#### 3.1.4. Statistical Analysis of CaA–ChA

As can be seen from Table 4, the IC_50_ mix for each ratio was lower than the IC_50_ add. The interaction index γ for all ratios of CaA–ChA was less than 1, suggesting that the CaA–ChA possessed synergistic antioxidant effects. In the tests for the free-radical-scavenging capacities, the CaA–ChA at a 1:1 mixture ratio demonstrated the optimal antioxidant capability, with an IC_50_ mix of 69.66 μg/mL for the DPPH assay and 21.65 μg/mL for the ABTS assay.

### 3.2. Screening of Key Targets and Pathways for Antioxidant Activity of CaA–ChA

#### 3.2.1. Targets of CaA–ChA Action

Using the TCMSP database, 9 targets related to CaA were identified. With the screening criteria of a “score > 20, *p* < 0.05”, 5 targets were selected using the BATMAN-TCM database. The Swiss Target Prediction database predicted 43 targets, and the Herb database queried 29 targets. After integration and de-duplication of the targets, a total of 73 targets for CaA were obtained. For ChA, 4 targets were found using the BATMAN-TCM database with the search conditions “score > 20, *p* < 0.05”. The Swiss Target Prediction database predicted 25 targets for ChA, and the Herb database found 6 targets. No targets related to ChA were found in the TCMSP database.

**Figure 3:**
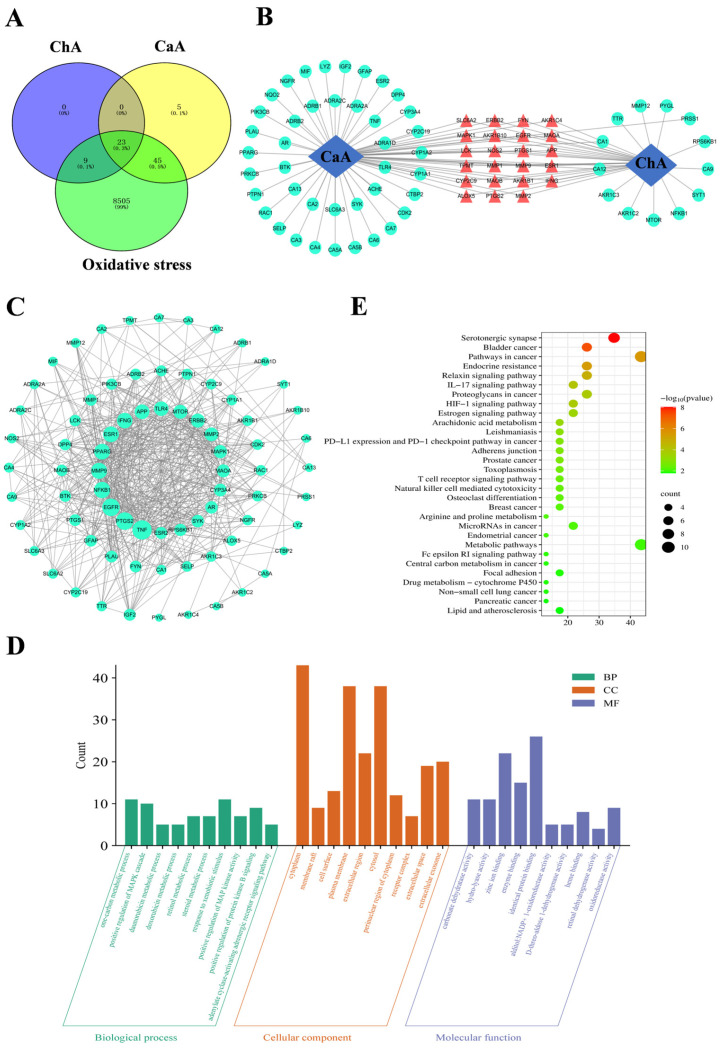
Network analysis of CaA–ChA. (**A**) The Venn diagram of the intersection targets. The blue circle represented the number of targets affected by ChA, the yellow circle represented the number of targets affected by CaA, and the green circle represented the number of targets related to oxidative stress. (**B**) Network diagram of targets of CaA–ChA and oxidative stress. The red triangle represents the common antioxidant targets of CaA–ChA, the green circle represents the antioxidant targets of CaA and ChA, respectively, and the gray line represented the interaction between the targets. (**C**) PPI network diagram of targets of CaA–ChA and oxidative stress. The larger the degree value, the larger the label. (**D**) GO function enrichment analysis results. (**E**) KEGG pathway enrichment analysis results.

A total of 8582 oxidative stress targets were obtained using the GeneCards and OMIM databases. Using the online tool Venny 2.1.0 to draw the intersection Venn diagram (Figure 3A), it could be seen that there were 68 intersection targets for CaA and oxidative stress, 32 intersection targets for ChA and oxidative stress, and 23 common antioxidant targets for both CaA and ChA.

#### 3.2.2. Construction of Targets Network and Protein–Protein Interaction (PPI) Network

Using Cytoscape 3.7.2 software to construct a network of targets for CaA, ChA, and oxidative stress, as shown in Figure 3B, the network contained a total of 79 nodes and 100 edges. Among them, the red targets were the antioxidant-related targets shared by ChA and CaA, with a total of 23, which might be the core targets for the synergistic antioxidant effects of CaA–ChA. Seventy-seven intersection targets were inputted into the software Cytoscape (3.7.2) to construct a network diagram. As shown in Figure 3C, the top five targets ranked by degree value in the network were TNF, PTGS2, EGFR, NFκB1, and MMP9.

#### 3.2.3. The GO Enrichment Analysis and KEGG Enrichment Analysis

The results of the GO enrichment analysis are shown in Figure 3D, with a total of 264 GO entries (*p* < 0.05). Among them, there were 223 biological process (BP) entries, including positive regulation of the MAPK cascade, response to xenobiotic stimulus, etc.; 32 cellular component (CC) entries, including cytoplasm, perinuclear region of the cytoplasm, extracellular exosomes, etc.; and 102 molecular function (MF) entries, including oxidoreductase activity, heme binding, etc.

The KEGG enrichment analysis results are presented in Figure 3E, and the results showed that the serotonergic synapse and the ROS signaling pathways were pathways directly related to antioxidation. The serotonergic synapse pathway played an important role in physiological functions such as learning, memory, emotion, motor function, and endocrine regulation [36]. Reactive oxygen species were significant factors in non-genetic carcinogenic pathways. Detoxification enzyme systems (such as cytochrome P450) produced a large amount of ROS while metabolizing xenobiotics (such as heavy metals, organic pollutants), leading to mutations in genetic material and consequently the occurrence of cancer.

### 3.3. The Antioxidant Effect of CaA–ChA in Zebrafish Intestine Tissue

#### 3.3.1. Effects of CaA–ChA on Intestinal Tissue Morphology

**Figure 4:**
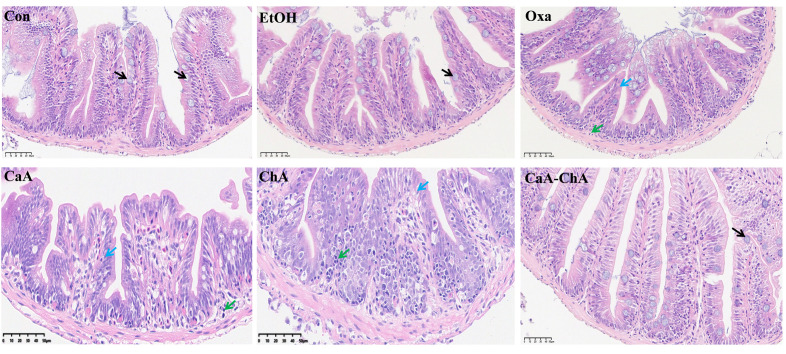
Effects of CaA–ChA on the intestinal tissue of zebrafish (black arrows indicate goblet cells, blue arrows indicate intestinal villus atrophy, and green arrows indicate inflammatory cell infiltration).

As shown in Figure 4, the intestinal tissue architecture of zebrafish in the blank control group was well-demarcated, consisting of the mucosa, muscularis externa, and serosa. The mucosal surface was covered with villi, each lined by a single layer of columnar epithelial cells, with abundant goblet cells scattered among these epithelial cells. No intestinal glands were identified in the lamina propria, and the muscularis externa exhibited a typical histoarchitectural pattern with inner circular and outer longitudinal muscle layers. No overt pathological lesions were observed in any of these layers. In comparison with the blank control group, the EtOH group also presented a clear intestinal tissue structure without detectable pathological alterations, indicating that EtOH, as the solvent used in this experiment, did not cause pathological damage to the intestinal tissue and that the interference of solvent factors with the experimental results could be excluded. After treatment with Oxa, the intestinal mucosal villi in the model group underwent atrophy with shortened lengths, while the basic structural integrity was retained. Meanwhile, the number of goblet cells was relatively reduced, and a marked infiltration of inflammatory cells was observed in the lamina propria. In the CaA group, the intestinal mucosal villi also showed atrophy and shortening, yet the structural clarity was preserved. A decrease in goblet cells counted and scattered inflammatory cell infiltration in the lamina propria were noted, with no other distinct pathological manifestations detected. Similar morphological changes were observed in the ChA group. The intestinal mucosal villi were atrophic and shortened with maintained structural integrity, accompanied by reduced goblet cell numbers and mild inflammatory cell infiltration in the lamina propria, without additional pathological changes. Notably, the intestinal tissue of zebrafish in the CaA–ChA group was restored to a normal state, with clearly delineated histologic layers and a normalized distribution of goblet cells.

#### 3.3.2. Effect of CaA–ChA on the mRNA Expression Levels of the Key Genes in the Intestine

**Figure 5:**
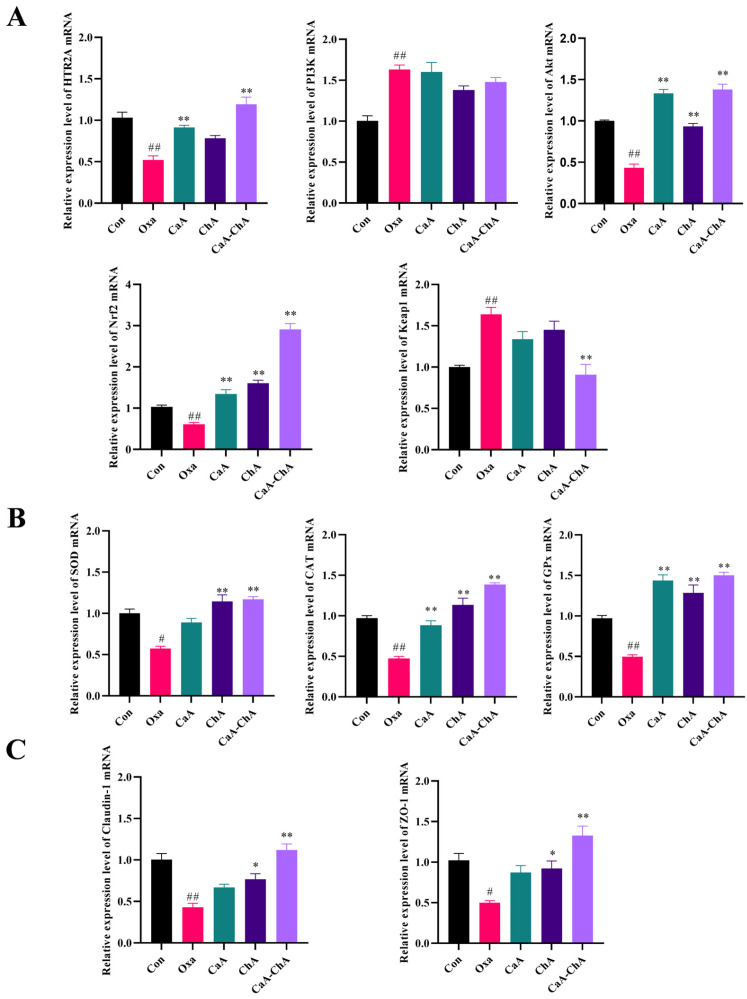
Effects of CaA–ChA on the intestine tissue of zebrafish. (**A**) Relative expression level of key gene mRNA in the HTR2A/Akt/Nrf2/Keap1 signaling pathway. (**B**) Relative expression level of antioxidant enzymes. (**C**) Relative expression level of tight junction proteins. The values were expressed as the mean ± SD (*n* = 3 per group). All data were analyzed using one-way analysis of variance (ANOVA) followed by Tukey’s post hoc test (compared with the control group, ^#^ *p* < 0.05, ^##^ *p* < 0.01; compared with the model group, * *p* < 0.05, ** *p* < 0.01).

Oxa treatment markedly perturbed the expression of key genes associated with the intestinal physiology and cellular signal transduction in zebrafish, whereas CaA–ChA intervention exerted a targeted regulatory effect. As shown in Figure 5A, compared with the blank control group, the intestinal *HTR2A* mRNA level in the Oxa group was significantly downregulated by approximately 50% (*p* < 0.01). This inhibitory effect was reversed upon treatment with CaA, ChA, and CaA–ChA, with statistically significant differences observed in the CaA and CaA–ChA groups (*p* < 0.01). The intestinal *PI3K* mRNA level was significantly elevated in the Oxa group relative to the blank control group (*p* < 0.01), while no notable alterations in *PI3K* mRNA expression were detected in the CaA, ChA, or CaA–ChA groups. Compared with the blank control group, the intestinal *Akt* mRNA level in the Oxa group was significantly decreased. Notably, CaA, ChA, and CaA–ChA treatments all led to a marked upregulation of *Akt* mRNA expression compared with the Oxa group (*p* < 0.01), with the highest expression level observed in the CaA–ChA group. Similarly, the intestinal *Nrf2* mRNA level in the Oxa group was significantly lower than that in the blank control group, whereas CaA, ChA, and CaA–ChA significantly elevated *Nrf2* mRNA expression compared with the Oxa group (*p* < 0.01), and the CaA–ChA group exhibited the peak expression level. In addition, the intestinal *Keap1* mRNA level was significantly increased in the Oxa group relative to the blank control group (*p* < 0.01). Of note, CaA–ChA treatment resulted in a significant reduction in *Keap1* mRNA expression compared with the Oxa group (*p* < 0.01), whereas CaA and ChA monotherapy failed to exert a significant inhibitory effect on *Keap1* mRNA expression.

#### 3.3.3. Effect of CaA–ChA on Oxidative Stress Biomarkers in Zebrafish Intestine Tissue

Oxa-induced intestinal injury was accompanied by a pronounced reduction in antioxidant enzyme expression, and this phenomenon was effectively alleviated by CaA–ChA treatment. As depicted in Figure 5B, compared with the blank control group, the intestinal *SOD* mRNA level in the Oxa group was significantly decreased (*p* < 0.01), indicating impaired ROS-scavenging capacity. In comparison with the Oxa group, the administration of CaA, ChA, and CaA–ChA all upregulated *SOD* mRNA expression (*p* < 0.01), with the ChA and CaA–ChA groups restoring *SOD* mRNA levels to values close to those of the control group. Consistent with the *SOD* expression profile, the intestinal *CAT* and *GPx* mRNA levels in the Oxa group were significantly downregulated compared with the blank control group (*p* < 0.01). All treatment groups exhibited a marked elevation in *CAT* and *GPx* mRNA levels relative to the Oxa group (*p* < 0.01), and the CaA–ChA combination treatment showed the most potent stimulatory effect on the expression of these two enzymes.

#### 3.3.4. Effect of CaA–ChA on Tight Junction Proteins in Zebrafish Intestine Tissue

Tight junction proteins are key molecules for maintaining intestinal epithelial barrier function, and their expression was significantly impaired after Oxa exposure, whereas CaA–ChA intervention exerted a protective effect on tight junction protein expression. As shown in Figure 5C, compared with the blank control group, the intestinal *Claudin-1* and *ZO-1* mRNA levels in the Oxa group were significantly reduced (*p* < 0.01), suggesting disruption of intestinal barrier integrity. Treatment with CaA, ChA, or CaA–ChA upregulated the expression of these two tight junction proteins, with the ChA and CaA–ChA groups showing statistically significant effects (*p* < 0.05). Collectively, the findings of the present study demonstrated that CaA–ChA combination treatment enhanced the function of the intestinal antioxidant defense system by upregulating the expression of *SOD*, *CAT*, and *GPx*, and its efficacy was superior to that of CaA–ChA monotherapy. This beneficial effect was associated with the upregulation of *HTR2A*, *Akt*, and *Nrf2* mRNA expression and downregulation of *Keap1* mRNA expression, which may contribute to the mitigation of Oxa-induced oxidative stress and intestinal mucosal injury.

#### 3.3.5. Effects of CaA–ChA on Intestinal Microbiota in Zebrafish

**Figure 6:**
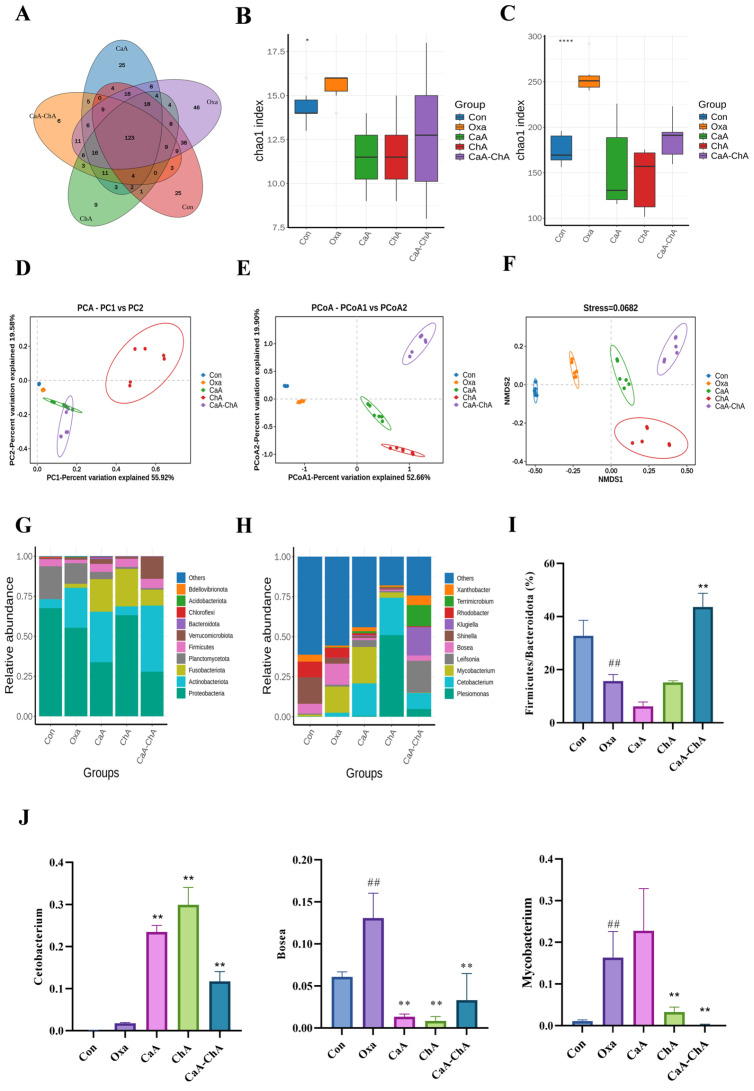
Effects of CaA–ChA on intestinal microbiota in zebrafish. (**A**) Species Venn diagram analysis. (**B**) Alpha diversity (Chao1) analysis at the phylum levels. (**C**) Alpha diversity (Chao1) analysis at the genus levels. (**D**) Principal component analysis (PCA). (**E**) Principal coordinate analysis (PCoA). (**F**) Non-metric multidimensional scaling (NMDS). (**G**) Species richness analysis at the phylum level. (**H**) Species richness analysis at the genus level. (**I**) The F/B ratio. (**J**) The relative abundances of *Cetobacterium*, *Bosea*, and *Mycobacterium*. All data were analyzed using one-way analysis of variance (ANOVA) followed by Tukey’s post hoc test (compared with the control group, ^##^ *p* < 0.01; compared with the model group, * *p* < 0.05, ** *p* < 0.01, **** *p* < 0.0001).

Following Oxa-induced intestinal injury and subsequent treatment with CaA, ChA, or CaA–ChA, the composition and structure of the intestinal microbiota in zebrafish were significantly remodeled (Figure 6). An analysis of unique operational taxonomic units (OTUs) showed that the number of unique OTUs in the control, Oxa, CaA, ChA, and CaA–ChA groups was 269, 331, 254, 221, and 221, respectively (Figure 6A). Compared with the control group, the Chao1 index at both the phylum and genus levels was significantly increased in the Oxa group (*p* < 0.05), suggesting that Oxa exposure abnormally enhanced the species richness of the intestinal microbiota. Conversely, CaA, ChA, and CaA–ChA interventions effectively restored the species richness of the zebrafish intestinal microbiota to near-normal levels (Figure 6B,C).

Results of α-diversity analysis indicated that at the phylum level, *Proteobacteria*, *Actinobacteriota*, *Fusobacteriota*, *Planctomycetota*, and *Firmicutes* were identified as the core dominant phyla in the zebrafish intestine. Relative to the control group, Oxa treatment significantly decreased the relative abundances of *Proteobacteria*, *Planctomycetota*, and *Firmicutes*, while remarkably elevating those of *Actinobacteriota* and *Fusobacteriota* (Figure 6G). Notably, Oxa exposure led to a prominent reduction in the ratio of *Firmicutes* to *Bacteroidota* (F/B ratio, *p* < 0.01), and this dysregulated pattern was significantly reversed by CaA–ChA intervention (*p* < 0.01). As a well-established indicator for evaluating intestinal metabolic function, a decreased F/B ratio is typically associated with an intestinal inflammatory status. These findings imply that Oxa-induced intestinal inflammation may be closely correlated with an F/B ratio imbalance, and CaA–ChA could ameliorate intestinal inflammatory responses by modulating this critical ratio (Figure 6I).

Further analysis of the abundance proportions of the top 10 genera (Figure 6H,J) demonstrated that Oxa treatment altered the native structure of the zebrafish intestinal microbiota and significantly increased the relative abundances of *Bosea* and *Mycobacterium* (*p* < 0.01). Interventions with CaA, ChA, and CaA–ChA all caused a significant reduction in *Bosea* abundance (*p* < 0.01). Specifically, both ChA and CaA–ChA treatments exerted a potent inhibitory effect on the excessive proliferation of *Mycobacterium* (*p* < 0.01). In addition, the relative abundance of *Cetobacterium* was significantly upregulated in the CaA, ChA, and CaA–ChA groups compared with the Oxa group (*p* < 0.01).

Results of β-diversity analysis revealed that the clustering patterns derived from principal component analysis (PCA), principal coordinate analysis (PCoA), and non-metric multidimensional scaling (NMDS) were highly consistent, with a distinct separation in the overall structure of the intestinal microbiota among different treatment groups (Figure 6D–F). This observation further confirmed that Oxa exposure and subsequent therapeutic interventions could exert a profound impact on the overall structure of the zebrafish intestinal microbiota.

#### 3.3.6. Correlation Analysis Between Oxidative-Stress-Related Biomarkers and Microbiota

**Figure 7:**
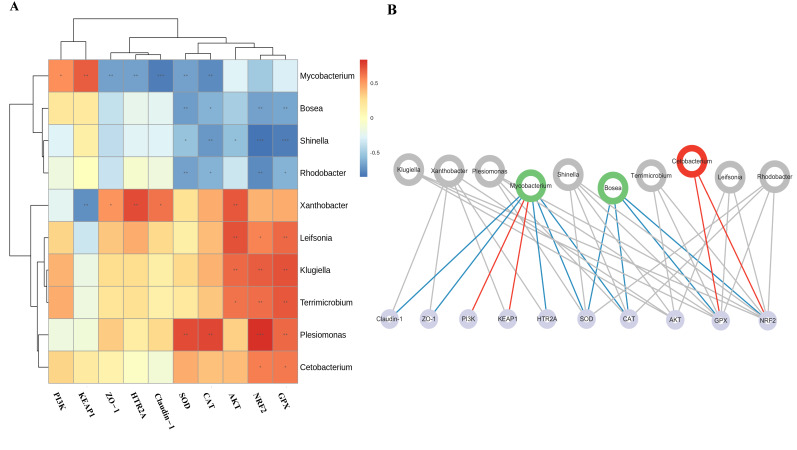
The correlation analysis between oxidative-stress-related biomarkers and intestinal microbiota. (**A**) Spearman analysis. The rows represented genera, and the columns represented environmental factors. The color of the blocks indicates the magnitude of the Spearman correlation coefficient, with red indicating a positive correlation and blue indicating a negative correlation. * *p* < 0.05, ** *p* < 0.01 and *** *p* < 0.001 represent a significant correlation. (**B**) Association network diagram between genus-level microorganisms and host functional genes. Red circles represent the genera with significantly increased abundance in the CaA–ChA group, and green circles represent the genera with significantly decreased abundance in the CaA–ChA group. Gray lines represent a correlation, red lines represent a positive correlation, and blue lines represent a negative correlation.

To predict the associations between oxidative-stress-related biomarkers (PI3K, KEAP1, ZO-1, HTR2A, Claudin-1, SOD, CAT, AKT, NRF2, and GPX) and intestinal bacteria, Spearman’s rank correlation analysis was performed at the genus level, with the results visualized in Figure 7A,B. The correlation analysis revealed that *Mycobacterium* exhibited a significant positive correlation with PI3K and KEAP1, whereas it was significantly negatively correlated with ZO-1, HTR2A, Claudin-1, SOD, and CAT. *Bosea* and *Rhodobacter* showed significant negative correlations with the antioxidant biomarkers SOD, CAT, NRF2, and GPX. *Shinella* was significantly negatively correlated with SOD, CAT, AKT, NRF2, and GPX. *Xanthobacter* presented a significant negative correlation with KEAP1, while displaying significant positive correlations with ZO-1, HTR2A, Claudin-1, and AKT. In contrast, *Leifsonia*, *Klugiella*, *and Terrimicrobium* were significantly positively correlated with AKT, NRF2, and GPX. *Plesiomonas* showed significant positive correlations with SOD, CAT, NRF2, and GPX, and *Cetobacterium* was significantly positively correlated with NRF2 and GPX. The above findings further corroborated the close correlations between the intestinal microbial genera *Cetobacterium*, *Bosea*, and *Mycobacterium*, as well as the key targets in the HTR2A/Akt/Nrf2/Keap1 pathway, antioxidant enzymes, and tight junction protein genes.

## 4. Discussion

**Figure 8:**
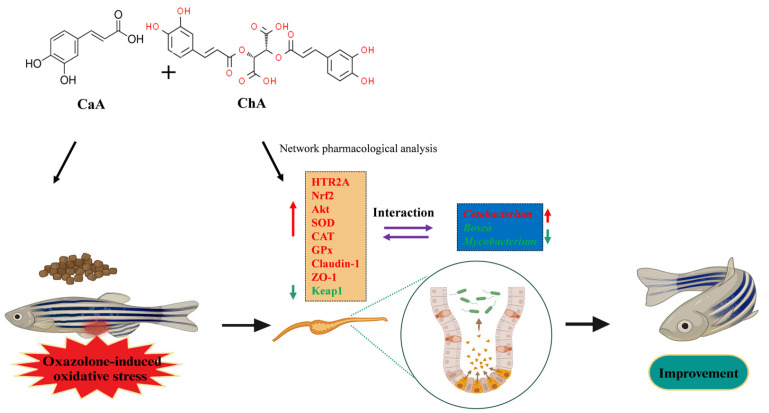
Mechanism diagram of the synergistic antioxidant effect of CaA–ChA (created in BioRender. Weiwei, Z. (2026) https://BioRender.com/f5tidg3, accessed on 19 March 2026. Red upward arrows represent upregulated gene expression or increased bacterial abundance; Green downward arrows represent downregulated gene expression or decreased bacterial abundance.).

Numerous previous studies have demonstrated that both CaA and ChA exhibit potent antioxidant activity, and their combined application with other polyphenolic compounds can elicit a synergistic antioxidant effect [37]. Meng et al. [38] employed a DPPH free-radical-scavenging assay and isobolographic analysis to investigate the synergistic antioxidant interaction between CaA and ChA. Their findings revealed that the effect point of the mixture fell below the 95% confidence interval, with an interaction index γ < 1, which indicated that the combination of CaA and ChA exerted a synergistic antioxidant effect. In line with these observations, the present study confirmed that the co-administration of CaA and ChA also exerted a synergistic effect on oxygen free radical scavenging. On this basis, the present study further selected the CaA–ChA as the research subject to elucidate the underlying mechanism of its antioxidant action (Figure 8).

In this study, we first evaluated the synergistic antioxidant effect of CaA and ChA and screened out their optimal combination ratio by means of an ABTS radical scavenging assay, DPPH radical scavenging assay, and isobolographic analysis. The results showed that the combined use of CaA and ChA significantly enhanced the antioxidant activity, and the IC_50_ of the complex was markedly lower than that of the two monomers acting alone. Specifically, when CaA and ChA were combined at a ratio of 1:1, both the IC_50_ value and interaction index of the complex reached the minimum, corresponding to the optimal synergistic antioxidant effect. Under this optimal ratio, the IC_50_ values of the complex for ABTS and DPPH radical scavenging were determined to be 21.65 μg/mL and 69.66 μg/mL, respectively.

Network pharmacology has been extensively applied in investigating the mechanisms of action of natural products. In the present study, a network pharmacology-based analytical strategy was employed to identify 77 overlapping targets associated with oxidative stress that were modulated by the CaA–ChA complex. Subsequent PPI analysis of these overlapping targets revealed that the top five core targets with the highest connectivity degrees were tumor necrosis factor (TNF), prostaglandin-endoperoxide synthase 2 (PTGS2), epidermal growth factor receptor (EGFR), nuclear factor kappa-B1 (NFκB1), and matrix metalloproteinase 9 (MMP9). Among these targets, TNF acts as a key pro-inflammatory cytokine involved in orchestrating inflammatory responses. Accumulating evidence has demonstrated that TNF-α inhibitors alleviate oxidative-stress-induced damage by suppressing lipid peroxidation, blocking the NFκB signaling pathway, and reducing the secretion of pro-inflammatory cytokines [39]. PTGS2 mediates the biosynthesis of prostaglandins in vivo and serves as a pivotal biomarker for oxidative stress [40]. NFκB1 exerts critical regulatory functions in both inflammatory responses and tumorigenesis [41]. Notably, the inhibition of NFκB activation in a cisplatin-induced renal injury model has been shown to promote the expression and nuclear translocation of Nrf2, thereby activating the endogenous antioxidant defense system [42]. MMP9 primarily functions in degrading the extracellular matrix (ECM). Studies using MMP9-knockout mice have indicated that the deficiency of MMP9 leads to a significant reduction in ROS levels and DNA damage in the intestinal tract, suggesting that MMP9 may ameliorate intestinal disorders by regulating ROS accumulation and DNA damage in the colon [43]. GO enrichment analysis indicated that the antioxidant effects of CaA–ChA were predominantly associated with biological processes including positive regulation of the mitogen-activated protein kinase (MAPK) cascade, cellular response to external stimulus, oxidoreductase activity, and heme binding. KEGG enrichment analysis further revealed that the synergistic antioxidant activity of CaA–ChA was closely correlated with the serotonergic synapse pathway, a well-recognized key signaling axis governing antioxidant capacity. The serotonergic synapse pathway is composed of serotonin (5-hydroxytryptamine, 5-HT) and its cognate receptors, whose components are widely expressed in intestinal tissues [44] and play essential roles in regulating multiple intestinal physiological functions, such as peristalsis, secretion, and sensory transduction [45]. Within this pathway, 5-hydroxytryptamine receptor 2A (HTR2A) is identified as a key target modulating the intestinal oxidative stress status. The activation of HTR2A has been verified to markedly reduce LPS-induced ROS production in intestinal epithelial cells, thereby ameliorating cellular oxidative stress [46]. Previous studies have confirmed a direct regulatory link between HTR2A and Akt. Specifically, knockdown of the HTR2A gene results in a significant decrease in Akt phosphorylation levels. Additionally, mounting evidence has established that the PI3K–Akt pathway directly modulates the Nrf2/Keap1 signaling axis, and impaired Akt expression leads to a notable inhibition of Nrf2 nuclear translocation [47]. Collectively, these network pharmacology findings suggested that the antioxidant effects of CaA–ChA were potentially mediated via the HTR2A/Akt/Nrf2/Keap1 signaling pathway.

Based on these network pharmacology predictions, in vivo validation experiments were further designed to elucidate the antioxidant efficacy and underlying mechanisms of CaA–ChA. Zebrafish were fed diets supplemented with CaA, ChA, or CaA–ChA (1:1 ratio) at a concentration of 0.03 mg/kg, respectively, to investigate the regulatory effects of CaA–ChA on key targets in the zebrafish intestine. RT-qPCR assays demonstrated that CaA–ChA treatment significantly upregulated the mRNA expression levels of *HTR2A*, *Akt*, and *Nrf2*, while concurrently downregulating *Keap1* mRNA expression in the zebrafish intestine [48,49,50]. Specifically, *Akt* expression was markedly decreased in the intestine of model group zebrafish, whereas its expression was significantly restored in the treatment groups, implying that Akt may serve as a core target mediating the antioxidant effects of CaA–ChA. Consistent with this hypothesis, existing literature has confirmed that Akt activation can trigger the Nrf2/Keap1 antioxidant signaling pathway [51]. Although CaA–ChA treatment promoted *PI3K* expression compared with the control group, no significant difference in *PI3K* expression was observed between the treatment groups and the model group. This finding suggested that PI3K might not be a core target involved in the antioxidant mechanism of CaA–ChA, and Akt activation was likely predominantly dependent on HTR2A-mediated phosphorylation modification rather than the transcriptional upregulation of *PI3K* [52]. These results collectively indicated that CaA–ChA exerted its antioxidant effects in association with regulation of the HTR2A/Akt/Nrf2/Keap1 pathway, which was consistent with the network pharmacology predictions. Building on these findings, the study further explored the regulatory effects of CaA–ChA on downstream antioxidant enzymes and tight junction proteins. The results showed that CaA–ChA treatment significantly ameliorated intestinal oxidative stress in zebrafish, as evidenced by the upregulated mRNA expression levels of *SOD*, *CAT*, and *GPx*. Concurrently, CaA–ChA enhanced the mRNA expression of the tight junction proteins *Claudin-1* and *ZO-1*, thereby exerting a protective effect on the intestinal barrier function. These results further confirmed that the antioxidant efficacy of the CaA–ChA complex was superior to that of the individual monomers, and its antioxidant activity was potentially associated with the modulation of the HTR2A/Akt/Nrf2/Keap1 signaling pathway. However, the present study only verified the association between CaA–ChA and the HTR2A/Akt/Nrf2/Keap1 pathway at the mRNA level. Further studies, such as western blot analysis for protein expression, Akt phosphorylation detection, an Nrf2 nuclear translocation assay, and pathway inhibition experiments, are still required to confirm the causal relationship between this pathway and the observed phenotypic changes.

To date, oxidative-stress-related studies have been conducted in a variety of animal models, including vertebrates, invertebrates, rodents, and fish. Among these models, zebrafish have emerged as a preferred vertebrate model organism for oxidative stress research due to their advantages such as low breeding costs, high genomic homology with humans, and short growth cycles [53]. The Oxa-induced zebrafish intestinal inflammation model is a classic experimental model for investigating intestinal immunity [54,55]. Our previous studies have successfully established the Oxa-induced zebrafish oxidative stress model and conducted investigations into the antioxidant activities of natural plant extracts based on this model [21]. Consistent with previous reports that the intestinal microbiota of normal adult zebrafish is primarily composed of phyla including *Proteobacteria*, *Actinobacteriota*, and *Fusobacteriota* [56,57], our sequencing results also verified this microbial composition profile. Notably, only CaA–ChA intervention could significantly reverse the Oxa-induced decrease in the F/B ratio. As a classic indicator for evaluating intestinal metabolic function, a reduced F/B ratio is typically associated with the intestinal inflammatory status [58]. Combined with the histopathological analysis results, these findings suggested that Oxa-induced intestinal inflammation might be closely correlated with an F/B ratio imbalance, and CaA–ChA exhibited superior antioxidant efficacy compared with individual CaA or ChA, which could ameliorate intestinal inflammatory responses by regulating the F/B ratio. Further analysis of microbial abundance at the genus level confirmed that CaA–ChA intervention significantly increased the abundance of *Cetobacterium* while reducing the abundances of *Bosea* and *Mycobacterium*. Previous studies have demonstrated that *Cetobacterium* is a beneficial gut bacterium in aquatic organisms such as fish, with core functions including vitamin B12 synthesis and nutrient metabolism; additionally, it can inhibit the colonization of pathogenic bacteria via nutrient competition [59]. Some strains of *Bosea* possess potential pathogenicity and may induce host inflammatory responses [60]. *Mycobacterium* encompasses multiple pathogenic species, and its excessive abundance can disrupt the intestinal microbial homeostasis, leading to intestinal barrier damage or inflammation [61]. These results confirmed that the combined intervention of CaA and ChA could positively regulate the intestinal microecological structure by selectively enriching the beneficial bacterium *Cetobacterium* and inhibiting the proliferation of the pathogenic bacteria *Bosea* and *Mycobacterium* in the zebrafish intestine.

Spearman correlation analysis revealed that the abundance of *Cetobacterium* was significantly positively correlated with the expression levels of *NRF2* and *GPX*. In contrast, *Bosea* exhibited a significant negative correlation with *SOD*, *CAT*, *NRF2*, and *GPX*. *Mycobacterium* was significantly positively correlated with *PI3K* and *KEAP1* but significantly negatively correlated with *ZO-1*, *HTR2A*, *Claudin-1*, *SOD*, and *CAT*. Collectively, these correlation data, combined with the characteristic changes in intestinal microbial abundance at the genus level (i.e., significant enrichment of *Cetobacterium* and significant reduction of *Bosea* and *Mycobacterium*) and the expression profiles of oxidative-stress-related indicators (i.e., significant upregulation of *HTR2A*, *AKT*, *NRF2*, *SOD*, *CAT*, *GPX*, *ZO-1*, and *Claudin-1*, along with significant downregulation of *KEAP1*), confirmed that *Cetobacterium*, *Bosea*, and *Mycobacterium* were closely associated with the antioxidant effect of CaA–ChA and may be potential target genera involved in the regulation of intestinal oxidative stress by CaA–ChA. However, the current microbiota analysis was correlative, and further studies (e.g., microbiota transplantation experiments, germ-free zebrafish models) are required to verify the causal relationship between these genera and the antioxidant effect of CaA–ChA.

Previous studies showed that *Sonchus brachyotus* DC. extract was rich in CaA and ChA, which significantly repaired oxidative damage via the Nrf2–Keap1–ARE signaling pathway at the cellular level and alleviated intestinal oxidative stress in Oxa-induced zebrafish by regulating the interaction between oxidative stress biomarkers and gut microbiota [20,21]. As natural phenolic acids, CaA and ChA follow typical plant polyphenol metabolic pathways in humans. Orally taken, CaA is slightly directly absorbed in the small intestine, while ChA requires hydrolysis by intestinal microbial esterases for absorption [62]. After absorption, they distribute to major tissues/organs and cross the blood–brain barrier [63]. The liver is the main metabolic organ, where they undergo phase I/II metabolism (mainly glucuronidation and sulfation) to form water-soluble metabolites [64], which are excreted via the kidneys with a 1–2 h elimination half-life [65]. Their metabolism is influenced by the intake dose, dietary composition, and intestinal microflora, to be further explored [66,67]. On this basis, the present study confirmed that CaA–ChA (1:1) exerted an antioxidant effect superior to single monomers. Dietary supplementation with 0.03 mg/kg CaA and 0.03 mg/kg ChA effectively alleviated zebrafish intestinal oxidative stress. Our previous study showed that the contents of CaA and ChA in the aqueous extract of *Sonchus brachyotus* DC. were 265 mg/g and 0.03 mg/g, respectively. Accordingly, when the extract was supplemented at ≥0.1% (1 g/kg feed), both CaA and ChA in the feed could simultaneously reach the effective level of 0.03 mg/kg, which was sufficient to exert a potential effect against intestinal oxidative stress. This study provides a theoretical basis for CaA–ChA as feed additives, offering support for developing high-efficiency, low-toxicity natural antioxidants.

## 5. Conclusions

This study adopted isobolographic analysis to investigate the synergistic antioxidant effect of the CaA–ChA combination, identify its optimal ratio, and quantify the corresponding antioxidant activity. Furthermore, an Oxa-induced zebrafish model of intestinal oxidative stress was established, and integrated analyses of molecular biology and intestinal microbiota were performed to elucidate the in vivo mechanism underlying the antioxidant action of CaA–ChA. The results confirmed that CaA and ChA exerted a significant synergistic antioxidant effect, with the strongest synergy achieved at a 1:1 ratio. At this optimal ratio, the IC_50_ values of the CaA–ChA mixture for scavenging ABTS and DPPH free radicals were 21.65 μg/mL and 69.66 μg/mL, respectively, demonstrating that its antioxidant activity was significantly superior to that of either monomer alone. In vivo validation experiments indicated that the CaA–ChA mixture exerts potent in vivo antioxidant activity, which is associated with the regulation of HTR2A/Akt/Nrf2/Keap1 pathway-related gene expression. Specifically, the CaA–ChA mixture significantly upregulated the mRNA expression levels of key genes (*HTR2A*, *Akt*, *Nrf2*) in this pathway while downregulating *Keap1* mRNA expression. This regulatory effect further activated the transcriptional expression of downstream antioxidant enzymes, including *SOD*, *CAT*, and *GPx*, thereby enhancing the intestinal antioxidant defense capacity. Meanwhile, the CaA–ChA mixture promoted mRNA expression of the tight junction proteins *Claudin-1* and *ZO-1*, which helped maintain intestinal barrier integrity and alleviate Oxa-induced intestinal damage. Intestinal microbiota analysis revealed that the CaA–ChA mixture could positively regulate the structure of the intestinal microecosystem in zebrafish, as evidenced by the targeted enrichment of the beneficial bacterium *Cetobacterium* and the significant inhibition of the proliferation of potential pathogenic bacteria (*Bosea* and *Mycobacterium*). Correlation analysis further verified that the abundance of *Cetobacterium* was significantly positively correlated with the expression of *NRF2* and *GPX*, whereas the abundances of *Bosea* and *Mycobacterium* were significantly negatively correlated with the expression of antioxidant enzyme genes (*SOD*, *CAT*, *GPX*) and intestinal-barrier-related genes (*ZO-1*, *Claudin-1*). These findings suggested that the interaction between intestinal microbiota and signaling pathway markers was involved in the regulation of the antioxidant process.

In summary, this study demonstrated that the CaA–ChA mixture (1:1 ratio) exerted significant antioxidant effects and protected against intestinal oxidative stress in Oxa-induced zebrafish. These beneficial effects were associated with the regulation of the relative abundances of core intestinal microbiota (*Cetobacterium*, *Bosea*, *Mycobacterium*) and the modulation of HTR2A/Akt/Nrf2/Keap1-pathway-related gene expression. These findings provide experimental and theoretical support for the combined antioxidative application of CaA and ChA.

## Figures and Tables

**Table 1:** Primer sequences.

Gene (Accession No.)		Primer Sequence (5′→3′)
*GAPDH* (NM_001115114.1)	F	TGCTGGTATTGCTCTCAACG
	R	GCCATCAGGTCACATACACG
*HTR2A *(XM_684208.11)	F	TACGGTGGCTGGGAACATTTTAG
	R	GGGACACAGTGATGCAGGGAAA
*PI3K* (NM_001281844.1)	F	ACATGGCTCTGCAAGATGCT
	R	GGAGGCATCTCGGACCAAAA
*Akt * (XM_073944553.1)	F	TCGGCAGGTGTCTTCTCAAT
	R	ACCCATTGCCATACCACGAG
*Nrf2 * (NM_182889.1)	F	GACAAAATCGGCGACAAAAT
	R	TTAGGCCATGTCCACACGTA
*Keap1 * (NM_182864.2)	F	TGATGGACAAACCCAACTCA
	R	CACTGGACAGGAAACCACCT
*SOD * (NM_131294.1)	F	GTCGTCTGGCTTGTGGAGTG
	R	TGTCAGCGGGCTAGTGCTT
*CAT * (NM_130912.3)	F	CAAGGTCTGGTCCCATAAA
	R	TGACTGGTAGTTGGAGGTAA
*GPx * (NM_001007281.2)	F	AGATGTCATTCCTGCACACG
	R	AAGGAGAAGCTTCCTCAGCC
*Claudin-1 * (XM_003448981.5)	F	CTTCACTCTGGTCGCCGTGTC
	R	GCAGCAAAGCATAGATCCTCCC
*ZO-1 * (XM_073943342.1)	F	CAGGGCGTCAAGAACATGAGG
	R	GTGGTGGTGAAAAGGTGATGG

**Table 2:** DPPH radical-scavenging rate of CaA–ChA at different ratios.

Mixture Ratio	Concentration (μg/mL)		Scavenging Rate (%)
	CaA	ChA	
1:2	12.5	25	7.14
	25	50	13.35
	50	100	23.72
	100	200	48.58
	150	300	70.09
1:1	12.5	12.5	19.51
	25	25	26.38
	50	50	41.46
	100	100	66.86
	150	150	90.23
2:1	25	12.5	8.08
	50	25	13.35
	100	50	23.78
	200	100	44.65
	300	150	66.76

**Table 3:** ABTS radical-scavenging rate of CaA–ChA at different ratios.

Mixture Ratio	Concentration (μg/mL)		Scavenging Rate (%)
	CaA	ChA	
1:2	2.5	5	3.53
	5	10	8.88
	10	20	16.00
	20	40	33.09
	30	60	49.36
1:1	2.5	2.5	4.66
	5	5	11.53
	10	10	23.12
	20	20	46.29
	30	30	69.28
2:1	5	2.5	3.92
	10	5	7.56
	20	10	17.67
	40	20	35.70
	60	30	53.86

**Table 4:** DPPH and ABTS scavenging rate of CaA–ChA.

	Mixture Ratio	IC_50add_ (μg/mL)	IC_50mix_ (μg/mL)	Interaction Index γ
ABTS	1:2	92.17	30.34	0.34
	1:1	88.17	21.65	0.25
	2:1	84.51	27.85	0.33
DPPH	1:2	332.44	105.3	0.32
	1:1	326.59	69.66	0.21
	2:1	320.94	111.4	0.35

## Data Availability

The original contributions presented in this study are included in the article. Further inquiries can be directed to the corresponding author. The gene accession number corresponding to the DNA sequences in the manuscript was PRJNA1423892.
